# Supplementary material for: Chirality-enhanced transport and drug delivery of graphene nanocarriers to tumor-like cellular spheroid
Source: Front Chem. 2023 Aug 2;11:1207579. doi: 10.3389/fchem.2023.1207579 (PMC10433752; doi:10.3389/fchem.2023.1207579)
Supplement: Supplementary file 1 [file DataSheet1.pdf]

Supplementary Material

Chirality-enhanced transport and drug delivery of graphene nanocarriers to tumor-like cellular spheroid

Hyunsu Jeon, Runyao Zhu, Gaeun Kim, Yichun Wang*

*** Correspondence:** Yichun Wang: ywang65@nd.edu

1 Supplementary Result

1.1 GQD Transport Estimation with Integrated Intensity Plot

To evaluate the chiral GQD transport into cellular aggregates and spheroids, we analyzed the integrated intensities of GQDs in the aggregate/spheroid regions (See **Materials and Methods Section 2.7**). For integrated intensity analysis, the GQD signal in the spheroid regions was collected and integrated throughout the spheroid area, showing the integrated intensity as a function of time (*i.e.*, $I_{total}(t)$; See equation (1) in **Materials and Methods Section 2.7**). **Supplementary Figure 4** shows the integrated intensity of each GQD within the cellular aggregate region (*e.g.*, 3 day-cultured cellular aggregates; *N*=3). As a result, *L*-GQD showed a faster intensity increase than *D*-GQD, indicating the stronger transport of *L*-GQD within the cellular aggregates. Similarly, **Supplementary Figure 8** shows the integrated intensity of each GQD within the cellular spheroid region (*e.g.*, 10 day-cultured cellular spheroids; *N*=3). As a result, integrated intensities of *L/D*-GQDs increased over time and reached each plateau, but their values differed significantly. Additionally, the times to reach a plateau of integrated intensities of *L/D*-GQDs were distinct: *L*-GQDs reached a plateau at nearly 20 min while that of *D*-GQD was 40 min, showing different observed times to reach the plateau. These facts imply the distinct differences between *L/D*-GQDs in their transport to tumor-like tissue.

2 Supplementary Figures and Tables

2.1 Supplementary Figures

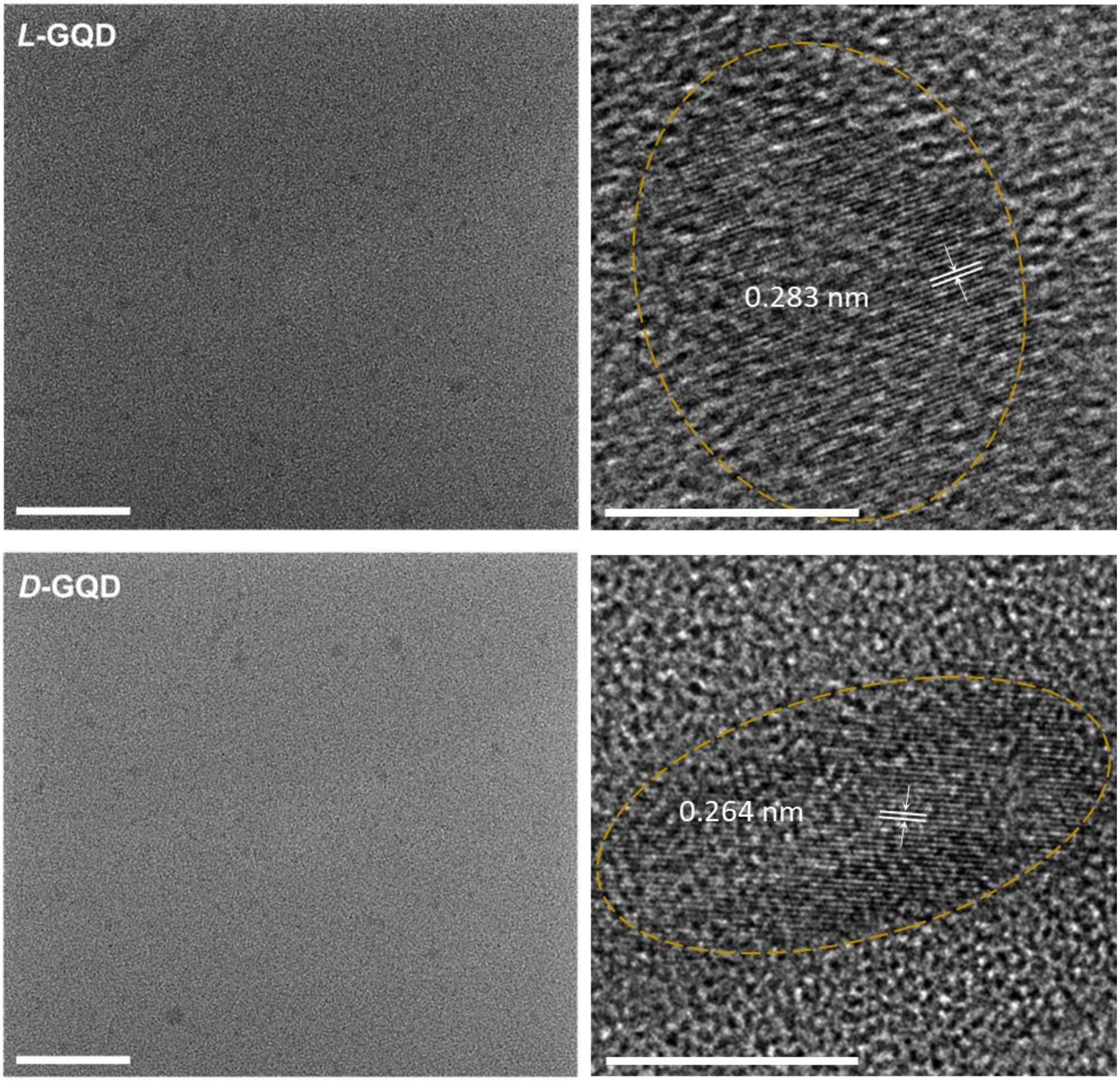

Supplementary Figure 1. Transmission electron microscopic (TEM) images of left/right-handed graphene quantum dots (*L/D*-GQDs) (Top: *L*-GQDs; Bottom: *D*-GQDs; Scale bar: 200 nm (left) and 10 nm (right)). Yellow dashed lines show the crystalline grid structure of *L/D*-GQDs.

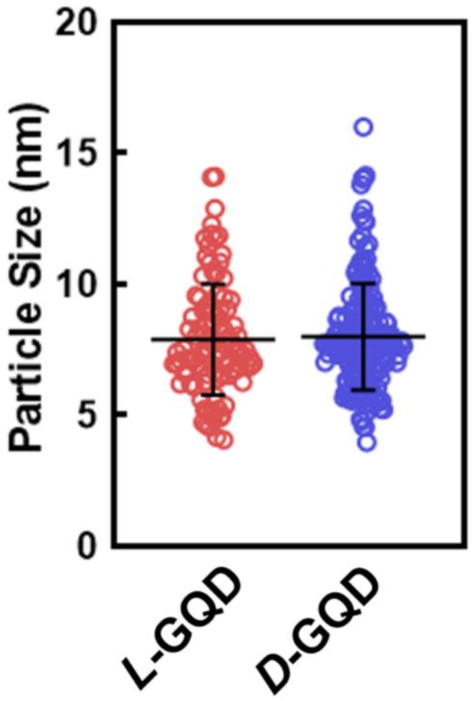

Supplementary Figure 2. (B) Size distribution plots for *L*/*D*-GQDs.

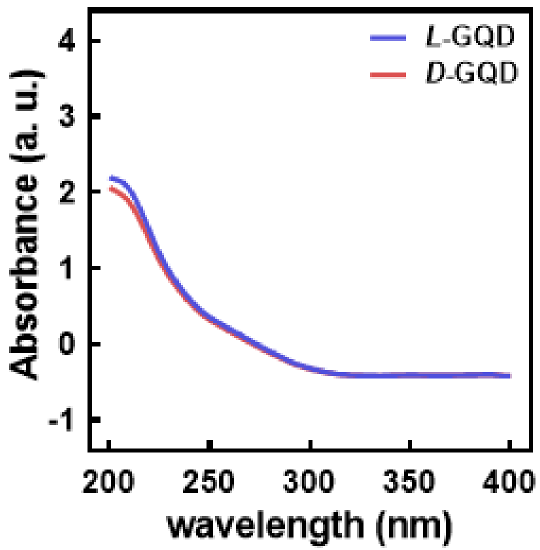

Supplementary Figure 3. Absorbance spectra of *L/D*-GQDs.

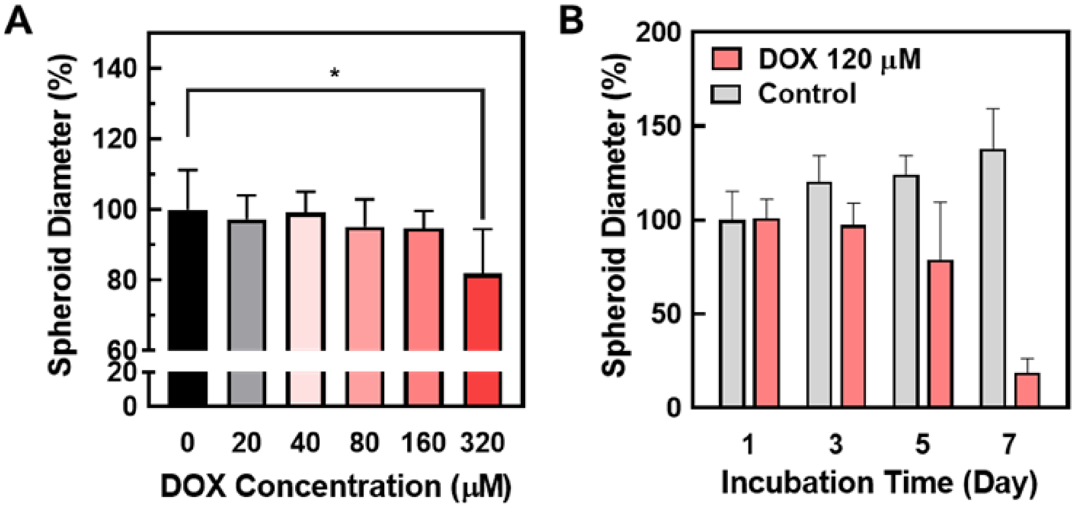

Supplementary Figure 4. Size change of cellular spheroids (*e.g.*, 10 day-cultured cellular spheroids) upon doxorubicin (DOX) treatment. **(A)** DOX concentration-dependent spheroid size changes. A range of DOX was treated to cellular spheroids for 6 h followed by 24 h incubation with fresh cell culture medium (*e.g.*, 0, 20, 40, 80, 160, and 320 μM, respectively; N=5; *: P<0.05). **(B)** DOX-induced size change of cellular spheroids upon increasing incubation time. 120 μM of DOX was treated to cellular spheroids for 6 h, followed by the size measurement at respective incubation times (*e.g.*, 24, 72, 120, and 144 h; N=5).

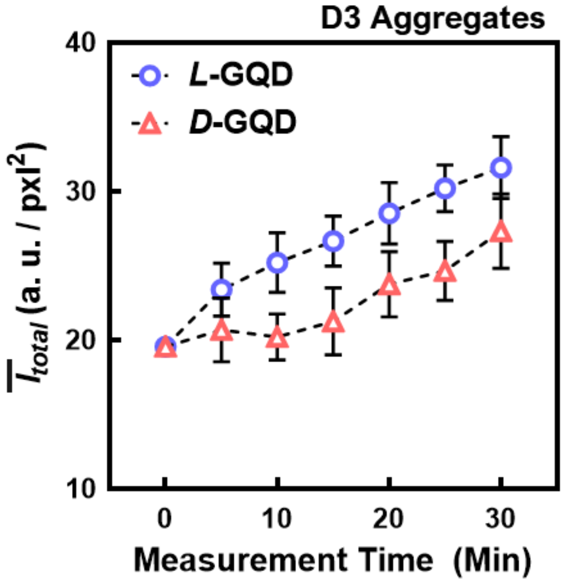

Supplementary Figure 5. Integrated GQD intensities increased within the region of cellular aggregates (*e.g.*, 3 day-cultured cellular aggregates) as a function of measurement time (min). Blue curves show the result from *L*-GQD-treated aggregates, while red curves show the result from *D*-GQD-treated aggregates.

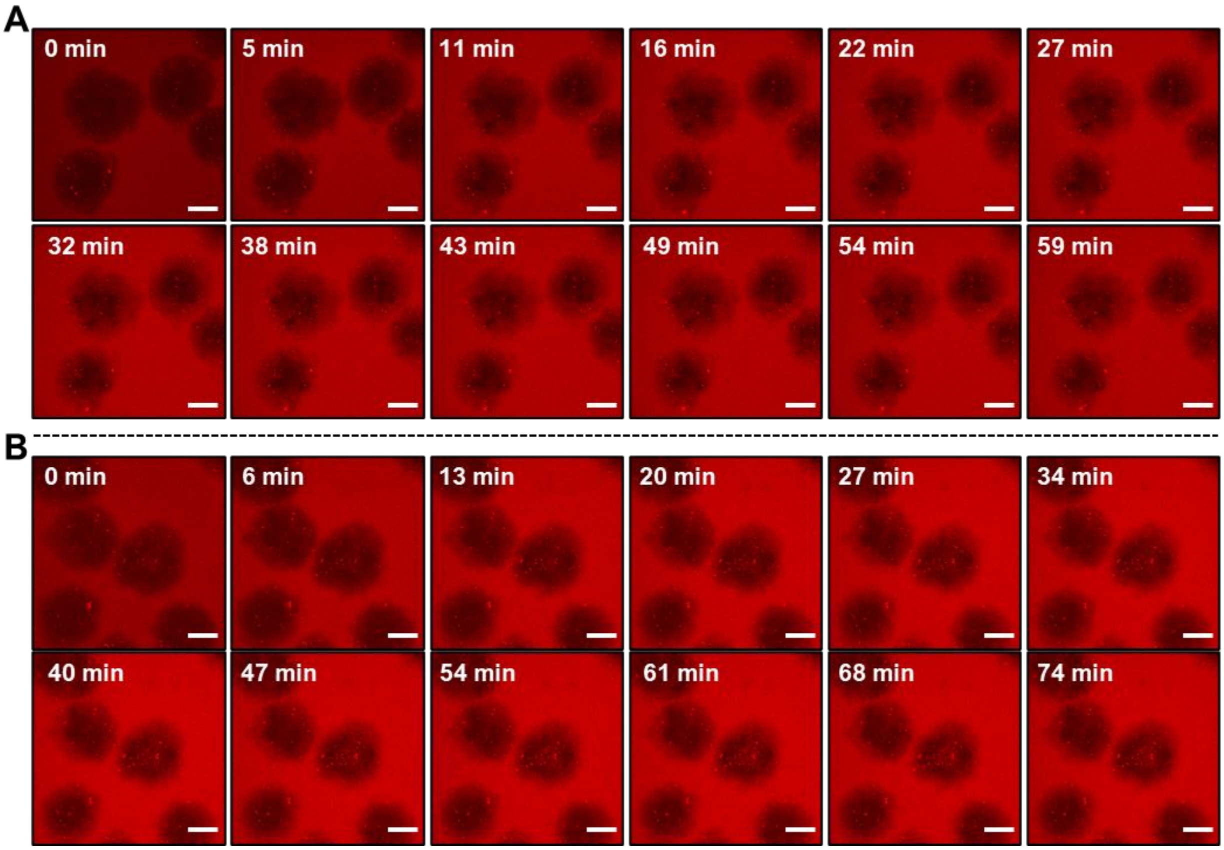

Supplementary Figure 6. Overall time-lapse confocal laser scanning electron microscopic (CLSM) images of GQD channel for *L*/*D*-GQD treatment to cellular spheroids. **(A)** The *L*-GQD transport monitoring. **(B)** The *D*-GQD transport monitoring (Scale bar: 200 μm).

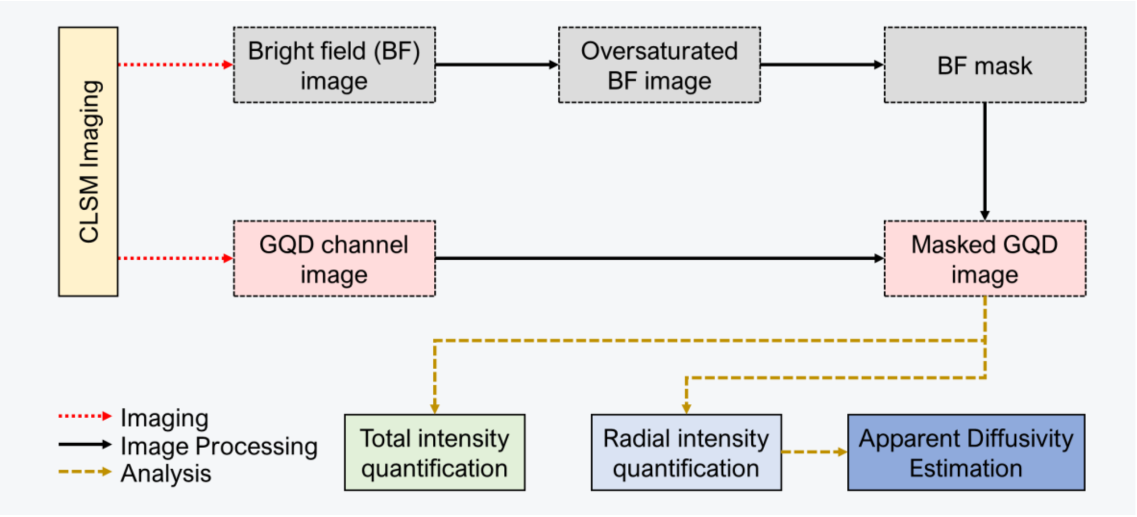

Supplementary Figure 7. The workflow for GQD signal collection and quantification from CLSM images.

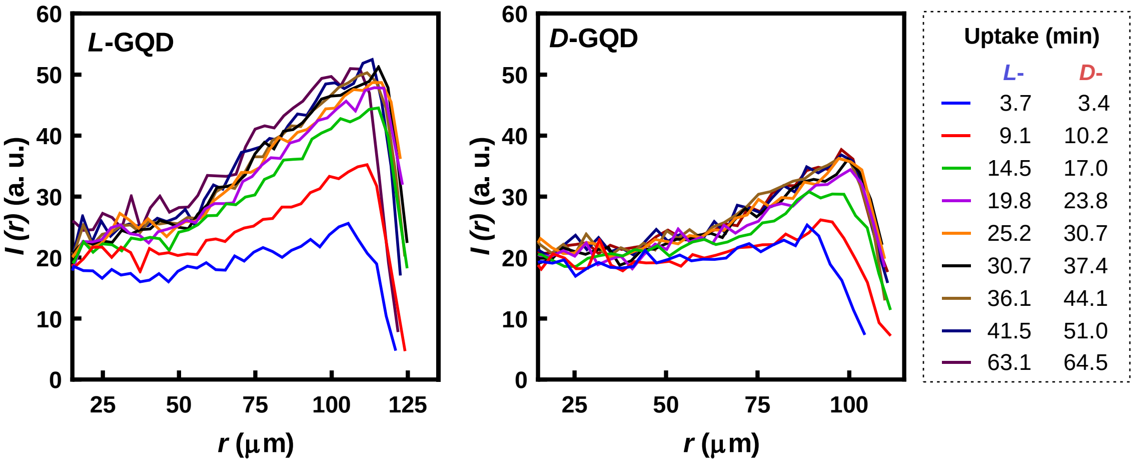

Supplementary Figure 8. The average GQD intensities as a function of radius of cellular spheroids under radial coordinates.

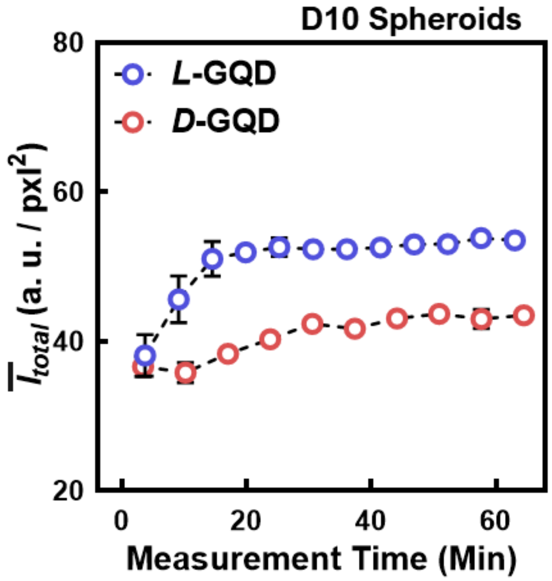

Supplementary Figure 9. Integrated GQD intensities increased within the region of cellular spheroids (*e.g.*, 10 day-cultured cellular aggregates) as a function of measurement time (min). Blue curves show the result from *L*-GQD-treated spheroids, while red curves show the result from *D*-GQD-treated spheroids.

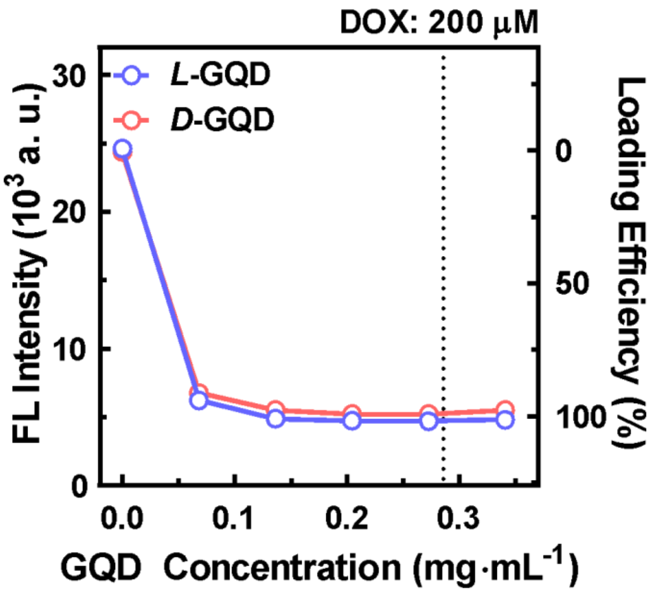

Supplementary Figure 10. Fluorescence-based DOX loading efficiency test using *L*/*D*-GQDs. The loading efficiency was determined by measuring DOX fluorescence at different *L*/*D*-GQD concentrations, which induced quenching of DOX. Fluorescence intensity was measured at 600 nm emission under 480 nm excitation.

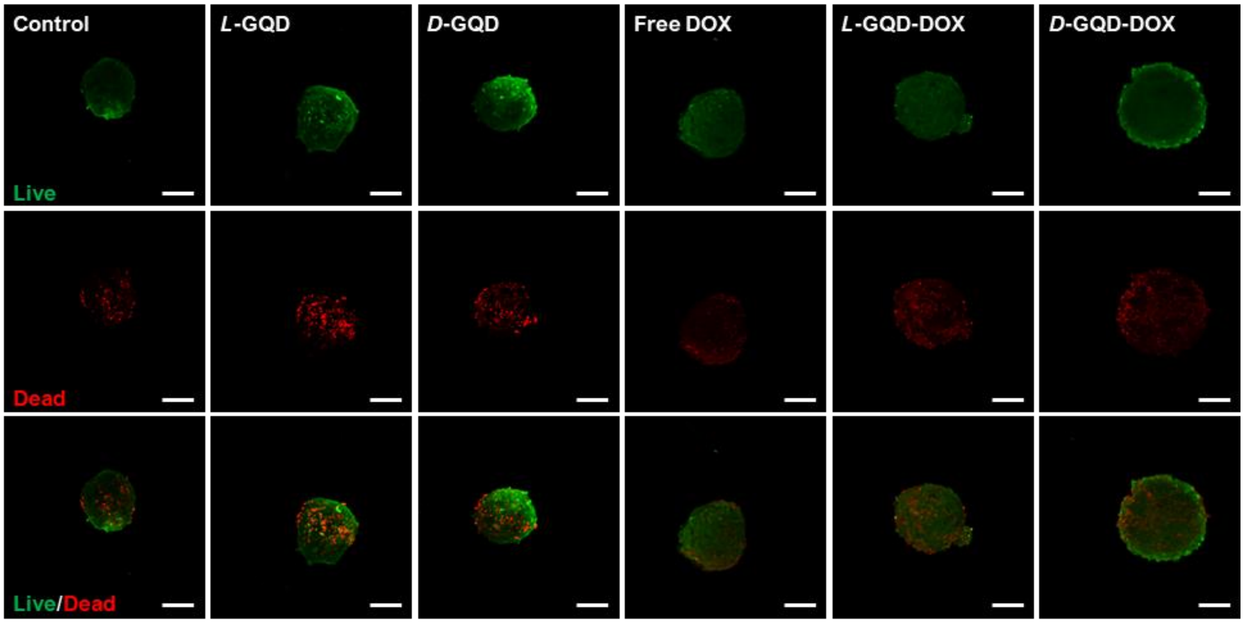

Supplementary Figure 11. The confocal fluorescent images of DOX-loaded *L/D*-GQD treatment to 3D cellular spheroid stained with Live/Dead assay (Green: live cell indicator, Red: dead cell indicator, Scale bar: 200 µm).

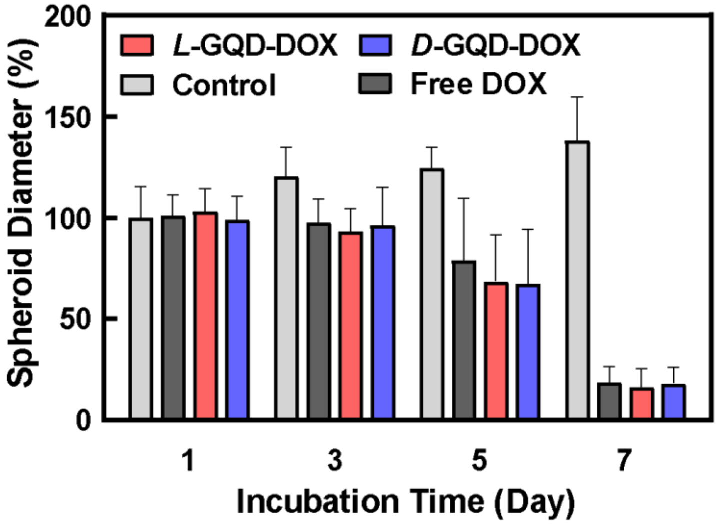

Supplementary Figure 12. Size change of cellular spheroids (*e.g.*, 10 day-cultured cellular spheroids) upon *L*/*D*-GQD-DOX treatment (*e.g.*, Prepared as GQD:DOX=0.5 mg·mL^{-1}:350 µM, and mixed with cell culture medium to 120 µM of DOX as final; See **Materials and Methods Section 2.2**). Free DOX-treated group (120 µM) was represented as the control group. The drugs were treated for 6 h followed by the size measurement at respective incubation times (*e.g.*, 24, 72, 120, and 144 h; *N*=5).

Supplementary Table 1. Wavenumbers of functional groups embedded in *L/D*-GQDs.

Functional Group	-OH	-C=O	-C=C	C-N	C-O
Wavenumber (cm^{-1})	3400<br>1409<br>1350	1712	1602	1250	1119
